# Determination of Exosome Mitochondrial DNA as a Biomarker of Renal Cancer Aggressiveness

**DOI:** 10.3390/cancers14010199

**Published:** 2021-12-31

**Authors:** Elena Arance, Viviana Ramírez, Alejandro Rubio-Roldan, Francisco M. Ocaña-Peinado, Catalina Romero-Cachinero, Ana Belén Jódar-Reyes, Fernando Vazquez-Alonso, Luis Javier Martinez-Gonzalez, Maria Jesus Alvarez-Cubero

**Affiliations:** 1 GENYO. Centre for Genomics and Oncological Research, Pfizer/University of Granada/Andalusian Regional Government, PTS Granada-Avenida de la Ilustración, 114-18016 Granada, Spain; elena.arance@genyo.es (E.A.); vivianarl@ugr.es (V.R.); alejandro.rubio@genyo.es (A.R.-R.); 2 Department of Statistics and Operations Research, University of Granada, 18071 Granada, Spain; fmocan@ugr.es; 3 Nursery Department. University Hospital Virgen de las Nieves, Av. de las Fuerzas Armadas 2, 18014 Granada, Spain; caroca23.cr@gmail.com; 4 Biocolloid and Fluid Physics Group, Excellence Research Unit Modeling Nature (MNat), Department of Applied Physics, School of Sciences, University of Granada, 18071 Granada, Spain; ajodar@ugr.es; 5 Urology Department, University Hospital Virgen de las Nieves, Av. de las Fuerzas Armadas 2, 18014 Granada, Spain; fvazquezalonso@gmail.com; 6 Department of Biochemistry and Molecular Biology III, Faculty of Medicine, PTS Granada, University of Granada, 18016 Granada, Spain; mjesusac@ugr.es; 7 Instituto de Investigación Biosanitaria ibs. GRANADA, 18014 Granada, Spain

**Keywords:** biomarker, extracellular vesicles, exosome, mitochondrial DNA, renal cancer

## Abstract

**Simple Summary:**

Components of liquid biopsy are potential non-invasive biomarkers for monitoring renal cell carcinoma (RCC) status. The aim of our study was to examine mitochondrial genes (such as *HV1* and *CYB*) included in exosomal fractions as promising and innovative biomarkers in RCC. We found that phase C containing different types of vesicles and phase F rich in exosomes with a high mitochondrial DNA (mtDNA) content could be considered as powerful biomarkers for susceptibility to RCC. Interestingly, mtDNA was a good genetic marker when aggressiveness was evaluated.

**Abstract:**

Here, the role of non-invasive biomarkers in liquid biopsy was evaluated, mainly in exosomes and mitochondrial DNA (mtDNA) as promising, novel, and stable biomarkers for renal cell carcinoma (RCC). A total of 140 fractions (named from B to F) obtained by ultracentrifugations of whole blood samples from 28 individuals (13 patients and 15 controls) were included. Nanoparticle Tracking Analysis (NTA) was conducted to characterized exosomal fraction. Subsequently, an analysis of digital PCR (dPCR) using the QuantStudio™ 3D Digital PCR platform was performed and the quantification of mtDNA copy number by QuantStudioTM 12K Flex Real-Time PCR System (qPCR) was developed. Moreover, Next Generation Sequencing (NGS) analyses were included using MiSeq system (Illumina, San Diego, CA, USA). An F fraction, which contains all exosome data and all mitochondrial markers, was identified in dPCR and qPCR with statistically significant power (adjusted *p* values ≤ 0.03) when comparing cases and controls. Moreover, present analysis in mtDNA showed a relevant significance in RCC aggressiveness. To sum up, this is the first time a relation between exosomal mtDNA markers and clinical management of RCC is analyzed. We suggest a promising strategy for future liquid biopsy RCC analysis, although more analysis should be performed prior to application in routine clinical practice.

## 1. Introduction

Renal cell carcinoma (RCC) is the third most common urologic malignancy, and remains one of the most lethal among urological ones [1]. The incidence of RCC is increasing globally, with rates varying by country, age, race, and sex [2]. Major troubles and sceneries in managing this disease are mainly: (i) Unspecific symptoms causing diagnosis in high stages; and (ii) Incidental detection of RCC by abdominal imaging techniques of new diagnosed tumors. These aspects reinforce the need of identifying novel predictive biomarkers for RCC diagnosis, progression, and prognosis [3]. RCC is not a single entity, but includes various tumor subtypes that have been identified on the basis of either characteristic pathologic features or distinctive molecular changes [4].

One of the major challenges of personalized oncology lies in identifying predictive biomarkers of response to therapy for their direct use in clinical setting [5]. For that reason, there are many efforts in searching biomarkers for a proper stratification that will help with an accurate treatment by the differentiation of diverse subtypes [6]. There are data that initiate a correlation between some expression patterns and a worse prognosis in clear-cell RCC (ccRCC), such as lower expression patterns in *AGXT, PTGER3*, and *SLC12A3*, or a reduced survival such as with higher expression patterns in *ALOX5* [7], or high expression patterns of *MXD3* as an independent risk factor for poor prognosis in ccRCC [8]. There are scarce data on the role of single nucleotide polymorphisms (SNPs) as biomarkers in RCC. It is well to name the role of rs10932384 (*ERBB4*), consistently associated with both recurrence and overall survival in stages I–III in RCC patients [9]. Several genes, such as *PAK1* and *PIK3R1*, have been found to be associated with a crucial role in cell migration and mobility in the RCC pathway by computational analysis data [10]. For example, it is reported that *PIK3R1* negatively regulated RCC migration and EMT (epithelial-mesenchymal transition) in vitro [11].

Others studies reported that over-expression of miR-15a was strongly associated with poor histological prognostic features of ccRCC, suggesting miR-15a as a potential prognostic molecular biomarker [12]. Target genes of miR-576 (*CUL3* and *RAC1*) have been identified to be involved in the regulation of multiple cancer-related biological pathways, and the target genes of miR-616 (*ASB13* and *FBXW2*) have been reported to be associated with the development of other cancers. Finally, these findings support that miR-576, miR-616 and miR-133a-2 may have guided significance for the early diagnosis of RCC [13]. Additionally, miR-103a-3p is suggested as a reflection of pathology and treatment response in other renal diseases [14].

In relation to the role of non-invasive biomarkers such as exosomes, increased patterns of Polymerase I and transcript release factor (PTRF)/Cavin1 detected in urine exosomes of ccRCC are suggested as biomarkers in this type of tumor [15]. Moreover, urinary exosomes are one of the best options. Recent studies suggest that changes in mRNA levels of urinary nano extracellular vesicles reflect the disease status of kidney tissues and their functional alterations [16]. Other studies correlate the role of miR-30c-5p in urinary exosomal fractions with ccRCC progression by *HSPA5* expression modulation [17]. Furthermore, recent reports indicate the full-length form of (pro)renin receptor ((P)RR), a single transmembrane protein encoded by the *ATP6AP2* gene, as a novel biomarker for cancer diagnosis, severity evaluation, and prognosis prediction. This is also a promising therapeutic target for cancer, including RCC [18].

Circulating cell-free tumor DNA (ccfDNA) is an interesting tool in the field of oncology. ccfDNA has been investigated as a potential biomarker in non-invasive diagnosis and prognosis, as well as disease monitoring. It can be easily isolated from bodily fluids or blood. Its origin is thought to be mainly from apoptotic or necrotic cell death, although active release mechanisms have also been suggested [19]. Numerous studies have confirmed that there is an elevated level of ccfDNA in the blood stream of cancer patients in comparison with healthy controls. These findings have opened up new possibilities for the development of clinical applications: Non-Invasive Prenatal Testing (NIPT), cancer diagnosis, transplantation medicine, and virology [20,21]. Valpione et al. [22] demonstrated the potential role of ccfDNA as a biomarker of tumor burden in metastatic melanoma patients, and that is a prognostic factor for overall survival. It is good to mention that ccfDNA includes nuclear DNA (nDNA) and mtDNA.

mtDNA exists as a circular and double-stranded nucleic acid with a high copy number. Variations in the copy number of circulating cell-free mtDNA (ccf-mtDNA) have been found in plasma and serum of patients with various tumors like breast, renal, ovarian, or lung [23,24]. The role of mtDNA and its susceptibility to oxidative stress and mutation is known [25], and, for that reason, there is a lot of new research focused on this molecule. There are reports indicating that mtDNA copy number variations in peripheral blood are associated with risk of developing several cancers such as colorectal cancer or pancreatic ductal adenocarcinoma. The association of mtDNA copy number with cancer risk has been studied in many cancer types with heterogeneous results. For example, in breast cancer, high mtDNA copy number was associated with a statistically significantly increased risk [26]; while in pancreatic ductal adenocarcinoma, the high level of mtDNA copy number was related with a reduced risk [27]. Recent data suggested mtDNA as a molecule with relevant prognostic value and staging in cancer [28].

To sum up, there are many biomarkers in clinical practice such as imaging molecular, magnetic resonance imaging, texture analysis, and radiomics or tissue biomarkers (Immunohistochemistry, mainly by the analysis of expression of *PAX8* and *PAX2*). However, there is much interest in determining serum (most of them proved in the *VEGF* pathways or *VHL* gene) and urine biomarkers (two urinary biomarkers that have shown evidence are the exosomal proteins aquaporin-1 (AQP-1) and perilipin-2 (PLIN2)) [6]. Here, we reinforced the role of non-invasive biomarkers in liquid biopsy, mainly in exosomes and mtDNA as promising, novel, and stable biomarkers that could improve current ones in RCC.

## 2. Materials and Methods

### 2.1. Patient Selection and Sample Collection

Participants enrolled in this study comprised the patient group (*n* = 13), and were recruited by urologists of “Virgen de las Nieves University Hospital”, Granada, Spain. Control (*n* = 15) blood samples were obtained from clinics “Gran Capitán, Salvador Caballero and Caseria de Montijo” of Granada. For more details, see Table 1. Controls were selected among healthy people with non-previous reports of tumors in their families and trying to have the same range of age and sex of patients collected for the present study. However, as can be seen in Table 1, there is a disparity in the number of males versus females among this selection. Samples were processed 4 h later from the collection. To obtain plasma, peripheral whole blood samples stored in ethylenediaminetetraacetic acid anticoagulant (EDTA) were centrifuged for 10 min at 1400 *g* and at 4 °C (more details in Figure S1). After separation, plasma samples were frozen at −80 °C until future analysis. The study protocol was approved by the Ethics Committee (CEI) with internal code 0165-N-19. Informed written consent from all participants was obtained in accordance with the tenets of the Declaration of Helsinki.

### 2.2. Isolation and Extraction of Exosomes from Plasma Samples

To carry out the exosomes purification, we started with 1 mL of plasma that had been previously frozen at −80 °C. In order to obtain different plasma fractions, successive centrifugations were carried out with the main aim of eliminating free part of exosomes containing apoptotic bodies and microvesicles (Table 2). Afterwards, an ultracentrifugation was performed (160,000× *g* at 4 °C for 2 h) for obtaining a purified fraction enriched in exosomes (phase F).

With the purpose of analyzing different plasma fractions, aliquots of 200 µL were taken from supernatants obtained after centrifugations (phases B and D). Sediment from the second centrifugation (phase C) was also analyzed, as well as the supernatant after ultracentrifugation (phase E), see details in Figure S1.

Therefore, a total of five aliquots corresponding to phases B to F were taken for each subject (*n* = 28), obtaining a total of 140 samples. All of them were kept at −20 °C until their subsequent DNA extraction. All these phases were concentrated at 43 °C (Thermo Scientific™ Savant™ DNA 120SpeedVac™ Concentrator). Followed by a DNA extraction carried out according to the method detailed by Freeman B et al. [29], a non-organic (proteinase K and salting out) protocol with some modifications described in Gomez-Martín et al. [30]. Subsequently, samples were quantified by Qubit Fluorometer and NanoDrop2000c systems (ThermoFisher Scientific, Waltham, MA, USA).

### 2.3. Characterization of Phase F by NanoSight LM10-HS

Particle concentration as a function of the diameter (hydrodynamic size distribution) was obtained by NTA (Nanoparticle Tracking Analysis). A NanoSight LM10 - HS (GB) FT14 (NanoSight, Amesbury, UK) equipped with a sample chamber, a 405 nm laser, and a high-sensitivity EMCCD (Electron Multiplying Charge-Couple Device) camera was used. Video images of the Brownian motion of the particles were captured and analyzed by the NTA v.2.3 image analysis software (Amesbury, UK). Two control and two patient samples (dilution 1/40) were measured at 25 °C and at least in triplicate, with manual shutter, gain, brightness, and threshold adjustments. Camera Level (CL) and Detection Threshold (DT) were fixed at 13 and 6, respectively.

### 2.4. Absolute Quantification of mtDNA in a Control Sample by Digital PCR (dPCR)

To determine the number of mtDNA copies in our quantified control DNA (qcDNA), we used two TaqMan^®^ probes (ThermoFisher Scientific, Waltham, MA, USA): TaqMan-FAM^TM^ (target gene, mtDNA_LP) and TaqMan-VIC^TM^ (endogenous gene, RNAse P). Probe sequence used for mtDNA_LP was 5′-TCGGCAAATCTTACCCC-3′. RNase P gene was run in the same PCR as an endogenous gene to determinate the target gene in each sample.

A five-fold dilution series were prepared to calculate the number of copies/µL in the control sample. Reactions were incubated at 96 °C for 10 min, followed by 39 cycles of 60 °C for 2 min, 98 °C for 30 s, and 60 °C for 2 min with a hold of 10 °C. A 9800 dual PCR System (ThermoFisher Scientific, Waltham, MA, USA) was used for the amplification. The QuantStudio™ 3D Digital PCR (dPCR) instrument was used to analyze the chips following the instructions in the “QuantStudio ™ 3D Digital PCR System User Guide” [31].

Then control sample was used as template for qPCR. A standard curve was constructed by plotting Ct (cycle threshold) values against the concentration of the mtDNA with different concentrations. The amount of mtDNA of patients and controls groups was quantified by interpolating Ct values in the standard curve.

### 2.5. Determination of the Relative Concentration of mtDNA by Real Time PCR (qPCR)

Quantification of mtDNA copy number was performed by QuantStudio^TM^ 12K Flex Real-Time PCR System (qPCR) (ThermoFisher Scientific, Waltham, MA, USA) according to the manufacturer’s protocol (iTaq™ Universal SYBR^®^ Green One-Step Kit, Bio-Rad, Hercules, CA, USA) [32].

Primers were designed for three different genetic regions: mitochondrial hypervariable region 1 (*HV1*), apocytochrome B of complex III (*MT-CYB*), and the hemoglobin subunit beta (*HBB*) as reference gene. In addition, two fragments of different length were designed: one of short size between 75 and 100 bp and another amplicon with a range of 175–200 bp for a long one. Primer sequences used for different regions are shown in Table S1. All these primers were used at a final concentration of 10 µM.

All reactions were incubated in a 96-well plate at 95 °C for 10 min, followed by 40 cycles of 95 °C for 15 s, and 60 °C for 1 min. All qPCR reactions were performed in triplicate and the negative controls (NTC) were included with every qPCR assay. To increase the statistical power, each replicate was deemed as an individual value. No amplification of the signal was observed when water was added instead of cDNA sample.

### 2.6. Next Generation Sequencing (NGS) Analyses and Data Processing

Extracted DNA was pooled from each of the phases belonging to both groups (controls and patients) and concentrated using the Concentrator plus (Eppendorf AG, Hamburgo, Germany). Libraries from DNA were prepared using between 0.5 and 20 ng of starting material and the KAPA HyperPrep Kit (Roche, Pleasanton, CA, USA) according to the manufacturer’s protocol until “hybridization of the amplified sample libraries and the SeqCap EZ probe pool” step. Concentration and quality of the Amplified Sample Library were measured using the Qubit 4 Fluorometer (ThermoFisher Scientific, Waltham, MA, USA) and the 2100 Bio-Analyzer Instrument (Agilent Technologies, Santa Clara, CA, USA).

Libraries were pooled in equal molar concentrations and sequenced on the MiSeq system (Illumina, San Diego, CA, USA) using MiSeq Reagent Kit v2 and paired-end 150 bp read lengths.

Raw and processed data quality controls were performed using FastQC and Qualimap tools. Reads were aligned using BWA v0.7.15 and Bowtiev2. Sam and Bam files were manipulated with Samtools v1.3.1. 

Reads per chromosome normalization were calculated, the formula of Reads per Kilobase Million (RPKM) is as follows:

(1) $$PKM=\frac{ER\;\times \;10^{9}}{EL\;\times MR}$$

We adapted the RPKM formula to provide a normalized measure of the number of reads that align with each chromosome based on their size: ER equals to the number of mapped read in each chromosome, EL to the chromosome length, and MR to the total number of mapped reads.

### 2.7. Statistical Analysis

SPSS v.26.0 (SPSS Inc., Chicago, IL, USA) was used for statistical analysis. Shapiro–Wilk’s test was performed to check the normality of the variables. Mann-Whitney test or Students *t*-tests analysis were used to check the differences in distribution of continuous variables. A multiple test correction was performed using the Benjamini and Hochberg technique, setting the False Discovery Rate (FDR) at 5%. The receiver operating characteristic (ROC) curve analysis was carried out to determine the diagnostic value of exosomal mtDNA copy number as a potential candidate biomarker for RCC. The area under the ROC curve (AUC) and 95% confidence interval (CI) was calculated. For all the statistical tests, the significance level was set at 0.03.

## 3. Results

### 3.1. NTA Analysis

**Figure 1:**
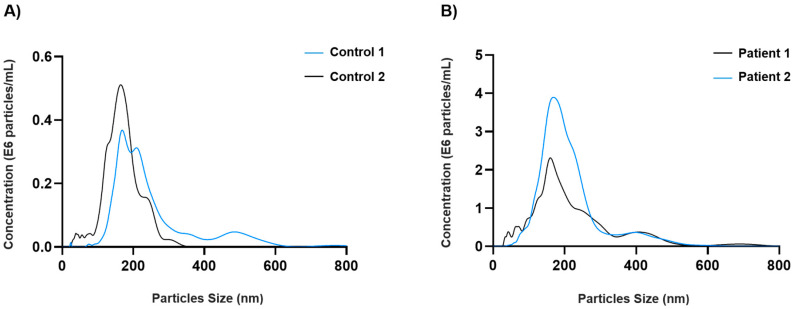
Size distribution and concentration of plasma exosomes by Nanoparticle Tracking Analysis (NTA). (**A**) Control 1 and 2 with a mean concentration of 0.497 and 0.503 × 10^8^ particles/mL, respectively. (**B**) Patient 1 (no metastasis) and patient 2 (metastasis) with a mean concentration of 3.493 and 5.170 × 10^8^ particles/mL, respectively.

The characterization of exosomal fractions from healthy participants and RCC patients was analyzed by NTA (Figure 1). The total concentration of particles expressed as 10^8^ particles/mL from 500 µL plasma was 0.5 ± 0.0047 in controls and 4.332 ± 1.185 in the patient group. The diameter of the particles expressed as the mean of the mode values was 173.5 ± 14.85 and 165.5 ± 5.4 in controls and patients, respectively.

### 3.2. mtDNA in a Control Sample by dPCR

We have compared both the Ct values and the parameters of copy number variation in mitochondrial and nuclear genes (Table S2). We used dPCR to see whether the different fractions (or phases) or genes are the most suitable or stable biomarkers in free DNA or extra vesicles fractions. As can be seen, in phase C, both genetic regions of *HV1* and *CYB* and the *HBB-short* gene showed statistical significance in qPCR analysis (adjusted *p* ≤ 0.001) and maintained when dPCR analyses were performed. Just *HBB-long* gene did not present statistical values in both analyses. Finally, phase F also performed relevant results according to mitochondrial genes as exosomes’ biomarkers showing remarkable significances both in qPCR and dPCR. By contrast, *HBB-short* and -long fragments did not show relevant results. As shown in Figure S2, we developed a bar chart representation to clarify that phases C and F were the most representative ones among the cases cohort.

**Figure 2:**
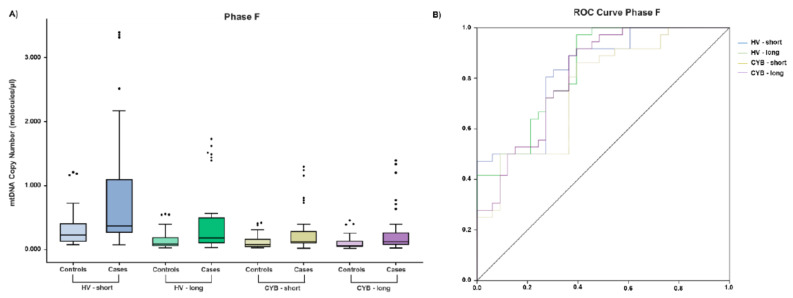
Exosomal mitochondrial biomarkers (**A**) Phase F comparison between cases and controls for mitochondrial hypervariable region 1 (*HV1*) and apocytochrome B (*CYB*) regions. (**B**) Receiver operating characteristic (ROC) curve analysis for mitochondrial markers.

Focusing in phase F, as shown in Figure 2A, it was nicely demonstrated that mitochondrial genes were representative markers among cases. Additionally, to determine the predictive ability of mtDNA copy number as biomarker to differentiate between controls and patients, a ROC curve analysis was developed. As seen in Figure 2B, *HV* (AUC = 0.825, 95% CI: 0.729–0.921, *p* < 0.0001 for *HV-short*, and AUC = 0.833, 95% CI: 0.740–0.927, *p* < 0.0001 for *HV-long*) and *CYB* (AUC = 0.755, 95% CI: 0.641–0.869, *p* < 0.0001 for *CYB-short*, and AUC = 0.810, 95% CI: 0.708–0.911, *p* < 0.0001 for *CYB-long*) genetic regions were good biomarkers for RCC.

### 3.3. mtDNA in Relation to Aggressiveness

According to aggressiveness, we developed an analysis to compare samples with higher aggressiveness in accordance with the presence of metastasis among the patient group. As can be seen in Table 3, both fragments of *HV1* presented significant values of aggressiveness in phase B by qPCR analysis, whereas copy number variation of *CYB-short* in phase C appeared to be a good biomarker for metastasis (adjusted *p* = 0.037). However, a greater significance was observed with *HBB-long* in most fractions in both analyses (qPCR and dPCR).

### 3.4. NGS Analyses

To evaluate the quality of mtDNA, NGS analyses were developed in all different samples. As can be seen in Table 4 by comparing data of RPKM and % mtDNA, phase C (0.361%), followed by B (0.038%), and finally F (0.016%) were the ones with a higher proportion of mtDNA.

When comparing reads alignment with Bowtie and BWA, we obtained the same results. Phase E and B are respectively the ones with the most efficient reads lectures, although all of them were about the 85% range recommended for libraries analysis. Moreover, phase C (in cases and controls) is the one with a higher proportion of mtDNA and vesicles (0.361%), following the same patterns as previously described in qPCR analysis. Furthermore, in phase F, we found a cover reads (0.016%) of mtDNA contained in vesicles with a high rate of mapping, as was also reported in qPCR.

## 4. Discussion

It is well known that the continuous improvements developed in clinical management of tumors are mainly focused on minimizing invasiveness of techniques like tissue biopsies. There is increasing research on improving liquid biopsies analysis. Most of them focus their attention on the analysis of CTCs (circulating tumor cells), cfDNA (cell free DNA), TEP (tumor educated platelets), or EVs (extracellular vesicles) [33]. Here, we proved that EVs are a good strategy as non-invasive biomarkers in RCC. It is known that EVs are vehicles of intercellular communication involved in many (patho) physiological processes. Moreover, these qualities joined to their molecular composition have positioned EVs as one of the most stable options for diagnostic and therapeutic purposes [34]. According to our data, phase F, which is the one harboring all exosomes, contains all mitochondrial markers totally stable in blood with significances among patients and controls, which makes them potential non-invasive biomarkers for RCC diagnosis. Furthermore, concerning NTA analysis, total concentration of exosomes was higher in patients than in controls, as expected. In accordance with a previous study, we obtained a similar size of exosomes, although the concentration of particles among patients was lower than that obtained by Pozo-Agundo et al. [35].

Moreover, we also discovered that phase C, which contains all mitochondrial fractions, is also another interesting marker for RCC diagnosis between cases and controls. Previous machine learning analysis developed in RCC based on RNA-sequencing found mitochondrial and angiogenesis-related genes signatures to be the most predictive ones within clustering approaches in clear cell, papillary, and chromophobe RCC. This analysis identified a high risk of ccRCC subgroup which is the best described by a mitochondrial signature and a down-regulation of angiogenesis-related genes, not exclusive to an RCC subgroup [36]. Even though the nuclear *HBB* region has shown remarkable significance in aggressiveness analysis, our data reinforced the power of mtDNA as an aggressiveness biomarker in RCC in metastasis.

Others metabolic genes, including *VHL, MTOR, ELOC, TSC1/2, FH, SDH*, as well as mtDNA, revealed the vast majority of RCC histology in the last 30 years [37]. On the other side, there are also nuclear markers, such as *FOXD1*, that have been suggested as a potent driver of tumor growth in ccRCC. *FOXD1* expression was inversely correlated with patient outcome and was also shown to be grade and stage dependent [38]. The role of mitochondrial damage in tumors is not new. For example, in ovarian cancer, MRPL15 (mitochondrial ribosomal protein 15) is suggested as a prognostic indicator and therapeutic target [39], or XRCC2 repairing mtDNA damage in hepatocellular carcinoma [40].

It is renowned that the detection of circulating EVs in the plasma of cancer patients represents a promising “liquid biopsy” strategy. Exosomes are the EVs in which more research is focused. Due to their multifactorial content, exosomes constitute a unique tool to capture the complexity and enormous heterogeneity of cancer in a longitudinal manner. Moreover, it is also due to molecular features like high nucleic acid concentrations and elevated coverage of genomic driver gene sequences [41]. Furthermore, recent studies developed by Lazar et al. [42] highlighted the possible role of platelet-derived microvesicles, as previously demonstrated in the role of platelets in cancer progression. In RCC, there are some studies which include the role of miRNAs in serum EVs as novel diagnostic markers. miRNA-4525 expression was higher in RCC tissue than in the adjacent normal tissue, suggesting this miRNA in EVs as a novel biomarker for RCC [43]. Exosomal miR-9–5p also plays an important role in RCC, indicating that it may be used as biomarker for diagnosis and monitoring the efficacy of therapy [44]. Concerning to metastatic ccRCC, an increase of EV-derived miR-301a-3p and a decrease of EV-derived miR-1293 is described [45]. Apart from miRNAs, EVs-derived tissue inhibitors of metalloproteinases (TIMP-1) mRNA are also included as good prognostic biomarker candidates for ccRCC [46]. In present work, we highlighted the role of EVs and focused on the strength of mtDNA as a relevant marker for both screening and aggressiveness.

The existence of fluctuation of copy number of mtDNA was previously reported in relation to injury and oxidative stress that contribute to the development of the toxicity of dioxin-like compounds [47] in acute myeloid leukemia (higher in aggressiveness stages) [48], hepatocellular [49], or tissue samples in breast cancer [50].

Mitochondria are considered as the power-generating units of the cell due to their key role in energy metabolism and cell signaling. For that reason, many studies concerning angiogenesis or other phases of cancer analyze them. Here, we focused on the findings of the stability of whole functional mitochondria in extracellular fluids like blood. These findings followed the same patterns as previous ones reported by Dache et al. [51], who detected extracellular full length mtDNA in particles over 0.22 μm holding specific mitochondrial membrane proteins in peripheral blood. Current efforts are mainly focused on the role of EVs; however, we demonstrated that mitochondria and mtDNA could also be a stable and potential analysis for cell-cell communication and cancer biomarker. We believe that circulating cell-free intact mitochondria have crucial biological and physiological roles because of their role as cell communication and hereditarian patterns [51]. This study is the second showing free structurally intact mitochondria in plasma, and the first indicating its role as stable molecule in RCC. Previously, Elsayed et al. [52] suggested that an increased peripheral blood mtDNA copy number is associated with increased risk of RCC. Therefore, RCC might be considered in the range of potential tumors in patients with an elevated blood mtDNA copy number. Here, we also indicated the most stable centrifugation conditions and phases (C and F) for mtDNA analysis, as well as the most suitable mitochondrial genetic markers for this purpose (*HV1* and *CYB*).

Our study has several limitations due to sample size, mainly by the difficulty in obtaining samples from RCC patients. NTA and NGS were also limited by this sample size. However, a high number of measurements were conducted for each sample when making ultracentrifugations.

## 5. Conclusions

We developed a simple and highly sensitive method that will permit detecting mtDNA variants as promise biomarkers in RCC. This experiment allowed us to analyze the fragment size distribution pattern of different regions of interest (as ccfDNA and content in EVs) in each plasma fraction (B to F), confirming the high mtDNA content in exosomes as a powerful biomarker. Therefore, application of liquid biopsy in the clinical scenario is a promising non-invasive technique for prediction, early diagnosis, and monitoring of cancer treatment. We affirm that it would be quite interesting to study how the amount of mtDNA varies in controls versus patients in fractions enriched in exosomal content, allowing the preservation of mtDNA thanks to its lipid bilayer structure. We have discovered that mitochondrial genes such as *HV1* and *CYB* were suitable markers for detecting aggressiveness and metastasis, with a high sensibility detected in qPCR and dPCR analysis and, for instance, in liquid biopsy. These new biomarkers will have relevant implications in diagnosis and management of RCC, where there are no current molecular biomarkers with direct applications in clinical scenarios. This is the first time that mtDNA is proposed as a stable biomarker in RCC and cancer management.

## Figures and Tables

**Table 1:** Characteristics of the study population.

Characteristic	Patients (*N* = 13)	Controls (*N* = 15)
Age (yr)		
Median (range)–yr	68 (47–88)	67 (44–93)
<65 yr	5	6
>65 yr	8	9
Sex		
Male	11	7
Female	2	8
Histology		
Papillary	1	NA
Clear cell	12	NA
Size tumor (cm)	9 (5–18)	NA
Stage		
Stage III	7	
Stage IV	6	
TNM		NA
T1	1	
T2	1	
T3	10	
T4	1	
Fuhrman nuclear grade		NA
G3	2	
G4	11	
Metastasis		NA
No	7	
Yes	6	

yr: years; NA: not applicable.

**Table 2:** Fractions obtained during exosomes collection.

Fraction	Sample	Obtaining
B	200 µL plasma	Plasma obtained after centrifugation (1400× *g*, 4 °C, 10 min)
C	Pellet	Pellet obtained after centrifugation with DTT + PBS (16,000× *g*, 4 °C, 20 min)
D	200 µL supernatant	Supernatant obtained after centrifugation (15,000× *g*, 4 °C, 30 min)
E	6 mL supernatant	Supernatant obtained after ultracentrifugation (160,000× *g*, 4 °C, 2 h)
F	Pellet	Pellet obtained after ultracentrifugation (160,000× *g*, 4 °C, 2 h)

**Table 3:** Representation of the values for risk of metastasis.

Phase	Gene	Adjusted *p* Value (*)	Adjusted *p* Value (cn)	Ct Mean No Metastasis ± SD	Ct Mean Metastasis ± SD	Copies Per µL Mean No Metastasis ± SD	Copies Per µL Mean Metastasis ± SD
B	*HV1-short*	*0.020*	0.069	24.54 ± 4.18	22.06 ± 2.08	2.38 ± 3.77	2.55 ± 2.04
	*HV1-long*	*0.035*	0.133	23.92 ± 4.61	21.35 ± 2.56	2.67 ± 3.92	2.56 ± 1.72
	*CYB-short*	0.078	0.223	25.71 ± 4.29	23.69 ± 2.54	2.08 ± 3.11	1.90 ± 1.35
	*HBB-long*	*0.020*	*0.029*	33.52 ± 2.99	36.09 ± 2.69	1.07 ± 2.02	0.09 ± 0.16
C	*CYB-short*	0.359	*0.037*	19.95 ± 2.73	20.67 ± 2.03	24.36 ± 29.66	10.18 ± 7.37
	*HBB-short*	*0.001*	*0.002*	19.18 ± 2.41	21.24 ± 0.89	1092.33 ± 1254.73	194.36 ± 102.38
	*HBB-long*	*0.006*	*0.012*	30.37 ± 3.71	32.99 ± 1.55	23.39 ± 49.39	0.38 ± 0.48
D	*HBB-long*	*0.001*	*0.001*	31.84 ± 3.48	36.03 ± 2.77	7.39 ± 15.66	0.07 ± 0.09
F	*HBB-long*	*0.007*	*0.014*	31.72 ± 2.13	33.44 ± 1.34	2.22 ± 4.42	0.22 ± 0.19

Ct: cycle threshold; *CYB*: Apocytochrome B; *HBB*: Hemoglobin subunit beta; *HV1*: Hypervariable region 1; SD: Standard deviation. HBB as nuclear marker vs. HV1 and CYB as mitochondrial markers. (*) Just FDR adjusted *p* values comparing genes in cases vs. controls by qPCR analysis. Adjusted *p* values (cn) represent values of comparisons cases vs. controls in copy number by dPCR analysis. The italics indicates significant *p* values.

**Table 4:** NGS analyses of the samples comparing autosomes versus mitochondrial genome.

Phase	% Mapped	Mapping Quality	Percentage in Genome	RPKM
B	99	28.65	Autosomes chr. 95.575% Sexual Chr. 4.387% mtDNA 0.038%	0.320 0.173 22.382
C	89	28.575	Autosomes chr. 95.331% Sexual Chr. 4.308% mtDNA 0.361%	0.316 0.179 212.287
D	98	29.055	Autosomes chr. 95.647% Sexual Chr. 4.344% mtDNA 0.010%	0.322 0.176 5.598
E	100	29.65	Autosomes chr. 95.665% Sexual Chr. 4.333% mtDNA 0.002%	0.319 0.177 0.437
F	94	29.535	Autosomes chr. 95.800% Sexual Chr. 4.184% mtDNA 0.016%	0.325 0.167 9.303

mtDNA: mitochondrial DNA; RPKM: Reads per Kilobase Million.

## Data Availability

Data is present in this article and Supplementary Materials.

## Supplementary Materials

The following supporting information can be downloaded at: https://www.mdpi.com/article/10.3390/cancers14010199/s1. Figure S1: Isolation and extraction of exosomes from plasma samples. Figure S2: Bar chart representation of mtDNA copy number variation of mitochondrial hypervariable region 1 (*HV1*) short gene among controls and cases comparing phases. Table S1: Sequences of designed primers. Table S2: Ct mean analysis cases vs. controls comparing genes and copy number.

## References

[1] Capitanio U., Bensalah K., Bex A., Boorjian S.A., Bray F., Coleman J., Gore J.L., Sun M., Wood C., Russo P. (2019). Epidemiology of Renal Cell Carcinoma. Eur. Urol..

[2] Meyer A.R., Allaf M.E., Gorin M.A., Gorin M.A., Allaf M.E. (2019). Epidemiology and Risk Factors of Renal Cell Carcinoma. Diagnosis and Surgical Management of Renal Tumors.

[3] Oto J., Plana E., Vicente Sanchez-Gonzalez J., Garcia-Olaverri J., Fernandez-Pardo A., Espana F., Martinez-Sarmiento M., Vera-Donoso C.D., Navarro S., Medina P. (2020). Urinary microRNAs: Looking for a New Tool in Diagnosis, Prognosis, and Monitoring of Renal Cancer. Curr. Urol. Rep..

[4] Signoretti S., Flaifel A., Chen Y., Reuter V.E. (2018). Renal Cell Carcinoma in the Era of Precision Medicine: From Molecular Pathology to Tissue-Based Biomarkers. J. Clin. Oncol..

[5] Dudani S., Savard M., Heng D.Y.C. (2020). An Update on Predictive Biomarkers in Metastatic Renal Cell Carcinoma. Eur. Urol. Focus.

[6] Farber N.J., Kim C.J., Modi P.K., Hon J.D., Sadimin E.T., Singer E.A. (2017). Renal Cell Carcinoma: The Search for a Reliable Biomarker. Transl. Cancer Res..

[7] Cui H., Shan H., Miao M.Z., Jiang Z., Meng Y., Chen R., Zhang L., Liu Y. (2020). Identification of the Key Genes and Pathways Involved in the Tumorigenesis and Prognosis of Kidney Renal Clear Cell Carcinoma. Sci. Rep..

[8] Zhang F., Liu L., Wu P., Li S., Wei D. (2021). Overexpression of MAX Dimerization Protein 3 (MXD3) Predicts Poor Prognosis in Clear Cell Renal Cell Carcinoma. Transl. Androl. Urol..

[9] Shu X., Gu J., Huang M., Tannir N.M., Matin S.F., Karam J.A., Wood C.G., Wu X., Ye Y. (2018). Germline Genetic Variants in Somatically Significantly Mutated Genes in Tumors Are Associated with Renal Cell Carcinoma Risk and Outcome. Carcinogenesis.

[10] Pido S., Ceddia G., Masseroli M. (2021). Computational Analysis of Fused Co-Expression Networks for the Identification of Candidate Cancer Gene Biomarkers. NPJ Syst. Biol. Appl..

[11] Lin Y., Yang Z., Xu A., Dong P., Huang Y., Liu H., Li F., Wang H., Xu Q., Wang Y. (2015). PIK3R1 Negatively Regulates the Epithelial-Mesenchymal Transition and Stem-Like Phenotype of Renal Cancer Cells through the AKT/GSK3β/CTNNB1 Signaling Pathway. Sci. Rep..

[12] Mytsyk Y., Borys Y., Tumanovska L., Stroy D., Kucher A., Gazdikova K., Rodrigo L., Kruzliak P., Prosecky R., Urdzik P. (2019). MicroRNA-15a Tissue Expression Is a Prognostic Marker for Survival in Patients with Clear Cell Renal Cell Carcinoma. Clin. Exp. Med..

[13] Tang T., Du X., Zhang X., Niu W., Li C., Tan J. (2019). Computational Identification and Analysis of Early Diagnostic Biomarkers for Kidney Cancer. J. Hum. Genet..

[14] Connor K.L., Denby L. (2021). MicroRNAs as Non-Invasive Biomarkers of Renal Disease. Nephrol. Dial. Transplant..

[15] Zhao Y., Wang Y., Zhao E., Tan Y., Geng B., Kang C., Li X. (2020). PTRF/CAVIN1, Regulated by SHC1 through the EGFR Pathway, Is Found in Urine Exosomes as a Potential Biomarker of ccRCC. Carcinogenesis.

[16] Fujitaka K., Murakami T., Takeuchi M., Kakimoto T., Mochida H., Arakawa K. (2021). mRNAs in Urinary Nano-Extracellular Vesicles as Potential Biomarkers for Non-Invasive Kidney Biopsy. Biomed. Rep..

[17] Song S., Long M., Yu G., Cheng Y., Yang Q., Liu J., Wang Y., Sheng J., Wang L., Wang Z. (2019). Urinary Exosome miR-30c-5p as a Biomarker of Clear Cell Renal Cell Carcinoma that Inhibits Progression by Targeting HSPA5. J. Cell. Mol. Med..

[18] Wang J., Nishiyama A., Matsuyama M., Wang Z., Yuan Y. (2020). The (Pro)Renin Receptor: A Novel Biomarker and Potential Therapeutic Target for Various Cancers. Cell Commun. Signal..

[19] Jahr S., Hentze H., Englisch S., Hardt D., Fackelmayer F.O., Hesch R.D., Knippers R. (2001). DNA Fragments in the Blood Plasma of Cancer Patients: Quantitations and Evidence for Their Origin from Apoptotic and Necrotic Cells. Cancer Res..

[20] Pan M., Chen P., Lu J., Liu Z., Jia E., Ge Q. (2020). The Fragmentation Patterns of Maternal Plasma Cell-Free DNA and Its Applications in Non-Invasive Prenatal Testing. Prenat. Diagn..

[21] Tsui N.B.Y., Jiang P., Chow K.C.K., Su X., Leung T.Y., Sun H., Chan K.C.A., Chiu R.W.K., Lo Y.M.D. (2012). High Resolution Size Analysis of Fetal DNA in the Urine of Pregnant Women by Paired-End Massively Parallel Sequencing. PLoS ONE.

[22] Valpione S., Gremel G., Mundra P., Middlehurst P., Galvani E., Girotti M.R., Lee R.J., Garner G., Dhomen N., Lorigan P.C. (2018). Plasma Total Cell-Free DNA (cfDNA) Is a Surrogate Biomarker for Tumour Burden and a Prognostic Biomarker for Survival in Metastatic Melanoma Patients. Eur. J. Cancer.

[23] Yu M. (2012). Circulating Cell-Free Mitochondrial DNA as a Novel Cancer Biomarker: Opportunities and Challenges. Mitochondrial DNA.

[24] Kohler C., Barekati Z., Radpour R., Zhong X.Y. (2011). Cell-Free DNA in the Circulation as a Potential Cancer Biomarker. Anticancer Res..

[25] Yang K., Li X., Forman M.R., Monahan P.O., Graham B.H., Joshi A., Song M., Hang D., Ogino S., Giovannucci E.L. (2019). Pre-Diagnostic Leukocyte Mitochondrial DNA Copy Number and Colorectal Cancer Risk. Carcinogenesis.

[26] Shen J., Platel M., Mahasheh A., Ambrosone C.B., Zhao H. (2010). Mitochondrial Copy Number and Risk of Breast Cancer: A Pilot Study. Mitochondrion.

[27] Gentiluomo M., Katzke V.A., Kaaks R., Tjonneland A., Severi G., Perduca V., Boutron-Ruault M., Weiderpass E., Ferrari P., Johnson T. (2020). Mitochondrial DNA Copy-Number Variation and Pancreatic Cancer Risk in the Prospective EPIC Cohort. Cancer Epidemiol. Biomark. Prev..

[28] Oliveira G.L., Coelho A.R., Marques R., Oliveira P.J. (2021). Cancer Cell Metabolism: Rewiring the Mitochondrial Hub. Biochim. Biophys. Acta-Mol. Basis Dis..

[29] Freeman B., Smith N., Curtis C., Huckett L., Mill J., Craig I.W. (2003). DNA from Buccal Swabs Recruited by Mail: Evaluation of Storage Effects on Long-Term Stability and Suitability for Multiplex Polymerase Chain Reaction Genotyping. Behav. Genet..

[30] Gomez-Martin A., Hernandez A.F., Javier Martinez-Gonzalez L., Gonzalez-Alzaga B., Rodriguez-Barranco M., Lopez-Flores I., Aguilar-Garduno C., Lacasana M. (2015). Polymorphisms of Pesticide-Metabolizing Genes in Children Living in Intensive Farming Communities. Chemosphere.

[31] ThermoFisher Scientific (2015). QuantStudio™ 3D Digital PCR System User Guide. https://assets.thermofisher.com/TFS-Assets/LSG/manuals/MAN0007720.pdf.

[32] Bio-Rad iTaq Universal Probes One-Step Kit, Vol. Mix, No. Table 1. https://www.bio-rad.com/webroot/web/pdf/lsr/literature/10032046.pdf.

[33] Carvalho A., Ferreira G., Seixas D., Guimaraes-Teixeira C., Henrique R., Monteiro F.J., Jeronimo C. (2021). Emerging Lab-on-a-Chip Approaches for Liquid Biopsy in Lung Cancer: Status in CTCs and ctDNA Research and Clinical Validation. Cancers.

[34] Ciferri M.C., Quarto R., Tasso R. (2021). Extracellular Vesicles as Biomarkers and Therapeutic Tools: From Pre-Clinical to Clinical Applications. Biology.

[35] Pozo-Agundo A., Villaescusa N., Martorell-Marugán J., Soriano O., Leyva S., Jódar-Reyes A.B., Botella L.M., Carmona-Sáez P., Blanco F.J. (2021). Identification of Exosomal MicroRNA Signature by Liquid Biopsy in Hereditary Hemorrhagic Telangiectasia Patients. Int. J. Mol. Sci..

[36] Marquardt A., Solimando A.G., Kerscher A., Bittrich M., Kalogirou C., Kubler H., Rosenwald A., Bargou R., Kollmannsberger P., Schilling B. (2021). Subgroup-Independent Mapping of Renal Cell Carcinoma-Machine Learning Reveals Prognostic Mitochondrial Gene Signature Beyond Histopathologic Boundaries. Front. Oncol..

[37] DiNatale R.G., Sanchez A., Hakimi A.A., Reznik E. (2020). Metabolomics Informs Common Patterns of Molecular Dysfunction Across Histologies of Renal Cell Carcinoma. Urol. Oncol. Semin. Orig. Investig..

[38] Bond K.H., Fetting J.L., Lary C.W., Emery I.F., Oxburgh L. (2021). FOXD1 Regulates Cell Division in Clear Cell Renal Cell Carcinoma. BMC Cancer.

[39] Xu H., Zou R., Li F., Liu J., Luan N., Wang S., Zhu L. (2021). MRPL15 Is a Novel Prognostic Biomarker and Therapeutic Target for Epithelial Ovarian Cancer. Cancer Med..

[40] Zhao Z., He K., Zhang Y., Hua X., Feng M., Zhao Z., Sun Y., Jiang Y., Xia Q. (2021). XRCC2 Repairs Mitochondrial DNA Damage and Fuels Malignant Behavior in Hepatocellular Carcinoma. Cancer Lett..

[41] Valencia K., Montuenga L.M. (2021). Exosomes in Liquid Biopsy: The Nanometric World in the Pursuit of Precision Oncology. Cancers.

[42] Lazar S., Goldfinger L.E. (2021). Platelets and Extracellular Vesicles and Their Cross Talk with Cancer. Blood.

[43] Muramatsu-Maekawa Y., Kawakami K., Fujita Y., Takai M., Kato D., Nakane K., Kato T., Tsuchiya T., Koie T., Miura Y. (2021). Profiling of Serum Extracellular Vesicles Reveals miRNA-4525 as a Potential Biomarker for Advanced Renal Cell Carcinoma. Cancer Genom. Proteom..

[44] Song W., Chen Y., Zhu G., Xie H., Yang Z., Li L. (2020). Exosome-Mediated miR-9-5p Promotes Proliferation and Migration of Renal Cancer Cells both In Vitro and In Vivo by Targeting SOCS4. Biochem. Biophys. Res. Commun..

[45] Dias F., Teixeira A.L., Nogueira I., Morais M., Maia J., Bodo C., Ferreira M., Silva A., Vilhena M., Lobo J. (2020). Extracellular Vesicles Enriched in Hsa-miR-301a-3p and Hsa-miR-1293 Dynamics in Clear Cell Renal Cell Carcinoma Patients: Potential Biomarkers of Metastatic Disease. Cancers.

[46] Dias F., Teixeira A.L., Nogueira I., Morais M., Maia J., Bodo C., Ferreira M., Vieira I., Silva J., Lobo J. (2020). Plasma Extracellular Vesicle-Derived TIMP-1 mRNA as a Prognostic Biomarker in Clear Cell Renal Cell Carcinoma: A Pilot Study. Int. J. Mol. Sci..

[47] VanEtten S.L., Bonner M.R., Ren X., Birnbaum L.S., Kostyniak P.J., Wang J., Olson J.R. (2021). Effect of Exposure to 2,3,7,8-Tetrachlorodibenzo-P-Dioxin (TCDD) and Polychlorinated Biphenyls (PCBs) on Mitochondrial DNA (mtDNA) Copy Number in Rats. Toxicology.

[48] Chaudhary S., Ganguly S., Palanichamy J.K., Singh A., Bakhshi R., Jain A., Chopra A., Bakhshi S. (2021). PGC1A Driven Enhanced Mitochondrial DNA Copy Number Predicts Outcome in Pediatric Acute Myeloid Leukemia. Mitochondrion.

[49] Liu Y., Zhou K., Guo S., Wang Y., Ji X., Yuan Q., Su L., Guo X., Gu X., Xing J. (2021). NGS-Based Accurate and Efficient Detection of Circulating Cell-Free Mitochondrial DNA in Cancer Patients. Mol. Ther.-Nucleic Acids.

[50] Rai N.K., Panjwani G., Ghosh A.K., Haque R., Sharma L.K. (2021). Analysis of Mitochondrial DNA Copy Number Variation in Blood and Tissue Samples of Metastatic Breast Cancer Patients (A Pilot Study). Biochem. Biophys. Rep..

[51] Dache Z.A.A., Otandault A., Tanos R., Pastor B., Meddeb R., Sanchez C., Arena G., Lasorsa L., Bennett A., Grange T. (2020). Blood Contains Circulating Cell-Free Respiratory Competent Mitochondria. FASEB J..

[52] Elsayed E.T., Hashad M.M., Elgohary I.E. (2017). Mitochondrial DNA Copy Number Variation as a Potential Predictor of Renal Cell Carcinoma. Int. J. Biol. Mark..

