# Supplementary material for: Determination of Exosome Mitochondrial DNA as a Biomarker of Renal Cancer Aggressiveness
Source: Cancers (Basel). 2021 Dec 31;14(1):199. doi: 10.3390/cancers14010199 (PMC8750318; doi:10.3390/cancers14010199)
Supplement: Supplementary file 1 [file cancers-14-00199-s001.zip › cancers-1517829-supplementary.pdf]

Supplementary Materials

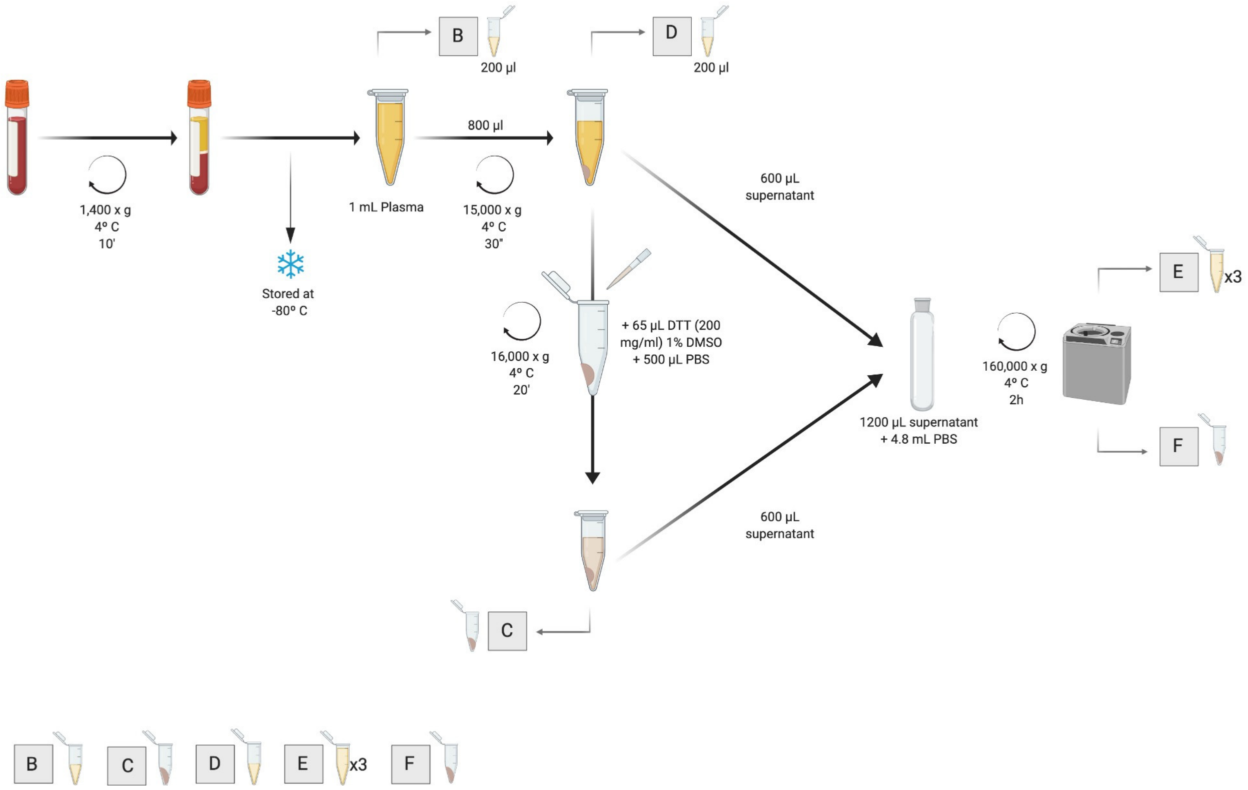

Figure S1. Isolation and extraction of exosomes from plasma samples where B-F are the different fractions analyzed.

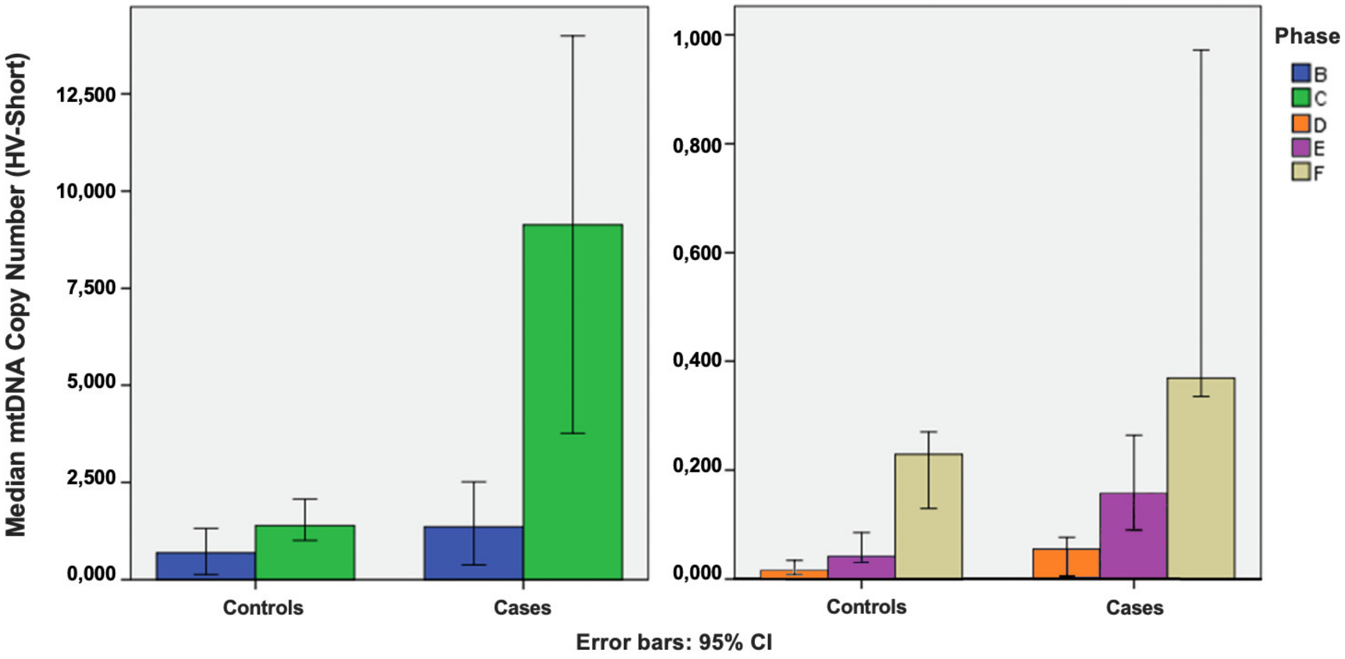

Figure S2. Bar chart representation of mtDNA copy number variation of mitochondrial hypervariable region 1 (*HV1*) short gene among controls and cases comparing phases.

Table S1. Sequences of designed primers**.**

Region	Short Fragment (75–100 pb)	Long Fragment (175–200 pb)
HBB	F: 5′-GCATAAAAGTCAGGGCAGAGC-3′<br>R: 5′-TGGTGTCTGTTTGAGGTTGCTA-3′<br>84 pb	F: 5′-ATTGCTTACATTTGCTTCTGACAC-3′<br>R: 5′-TGGTCTCCTTAAACCTGTCTTGTA-3′<br>187 pb
HV1	F: 5′-AGTACATAAAAACCCAATCCACATC-3′<br>R: 5′-GATGTGTGATAGTTGAGGGTTGAT-3′<br>85 pb	F: 5′-TACATTACTGCCAGCCACCA-3′<br>R: 5′-GTTGGTATCCTAGTGGGTGAGG-3′<br>186 pb
CYB	F: 5′-CCACATCACTCGAGACGTAAAT-3′<br>R: 5′-GGAAGAGGCAGATAAAGAATATTGA-3′<br>86 pb	F: 5′-CTAGCCATGCACTACTCACCAG-3′<br>R: 5′-TTTCTGAGTAGAGAAATGATCCGTAA-3′<br>184 pb

CYB: Apocytochrome B; *HBB:* Hemoglobin subunit beta; *HV1:* Hipervariable región 1; F: forward primer; R: reverse primer.

Table S2. Ct mean analysis cases vs controls comparing genes and copy number.

Phase	Gene	Adjusted p Value (*)	Adjusted p Value(cn)	Ct Mean Controls ± SD	Ct Mean Patients ± SD	Copies per µl Mean Controls ± SD	Copies per µl Mean Patients ± SD
B	*HV1-short*	0.084	0.064	24.79 ± 3.58	23.42 ± 3.57	1.09 ± 1.18	2.46 ± 3.06
	HV1-long	*0.013*	*0.008*	25.10 ± 4.46	22.73 ± 3.97	0.90 ± 1.18	2.62 ± 3.07
	CYB-short	*0.043*	*0.007*	26.43 ± 3.67	24.77 ± 3.69	0.69 ± 0.72	2.00 ± 2.43
	CYB-long	*0.014*	*0.003*	27.80 ± 4.91	25.11 ± 4.86	0.48 ± 0.65	1.67 ± 1.82
	HBB-short	0.512	0.245	32.47 ± 2.29	32.82 ± 2.51	0.62 ± 0.85	0.76 ± 1.32
	HBB-long	0.213	0.205	35.72 ± 3.02	34.80 ± 3.09	0.56 ± 1.91	0.58 ± 1.49
C	*HV1-short*	*<0.001*	*0.001*	21.62 ± 2.39	19.48 ± 2.32	5.93 ± 10.04	18.73 ± 32.15
	HV1-long	*<0.001*	*0.001*	20.41 ± 2.55	18.30 ± 2.43	7.81 ± 13.50	24.54 ± 45.83
	CYB-short	*0.001*	*0.002*	22.34 ± 2.79	20.28 ± 2.43	7.95 ± 12.76	17.82 ± 23.21
	CYB-long	*0.001*	*0.002*	21.69 ± 2.98	19.64 ± 2.52	8.29 ± 13.60	18.75 ± 27.54
	HBB-short	*<0.001*	*<0.001*	21.74 ± 1.73	20.13 ± 2.12	209.85 ± 229.09	677.88 ± 1019.3
	HBB-long	0.102	0.163	32.46 ± 2.42	31.58 ± 3.17	1.48 ± 3.26	12.77 ± 37.67
D	*HV1-short*	0.783	0.932	29.37 ± 2.64	29.20 ± 2.68	0.047 ± 0.06	0.046 ± 0.04
	HV1-long	*0.024*	*0.003*	29.33 ± 2.90	27.83 ± 3.06	0.08 ± 0.24	0.11 ± 0.18
	CYB-short	*0.002*	*0.003*	32.72 ± 3.46	30.38 ± 3.31	0.022 ± 0.04	0.07 ± 0.08
	CYB-long	*0.001*	*0.001*	33.91 ± 4.88	30.49 ± 3.69	0.043 ± 0.11	0.05 ± 0.07
	HBB-short	0.050	0.068	34.72 ± 2.83	33.39 ± 3.16	0.25 ± 0.44	0.66 ± 1.04
	HBB-long	0.209	0.254	35.22 ± 3.47	34.12 ± 3.72	0.69 ± 1.96	3.40 ± 10.99
E	*HV1-short*	0.068	*0.026*	19.82 ± 12.38	24.04 ± 7.54	0.39 ± 0.75	0.36 ± 0.47
	HV1-long	*0.020*	*0.027*	26.84 ± 3.24	25.34 ± 1.96	0.27 ± 0.64	0.27 ± 0.37
	CYB-short	*0.002*	*0.003*	27.96 ± 3.96	25.34 ± 2.45	0.28 ± 0.38	1.09 ± 1.64
	CYB-long	0.750	0.413	34.34 ± 3.36	34.70 ± 4.87	0.005 ± 0.009	0.05 ± 1.78
	HBB-short	0.721	0.564	31.58 ± 2.96	31.86 ± 3.44	0.92 ± 0.57	1.47 ± 2.02
	HBB-long	*0.012*	*0.012*	33.93 ± 3.17	35.91 ± 2.71	0.75 ± 1.76	0.09 ± 0.13
F	*HV1-short*	*<0.001*	*<0.001*	25.16 ± 1.29	23.79 ± 1.62	0.32 ± 0.29	0.87 ± 0.92
	HV1-long	*<0.001*	*<0.001*	25.82 ± 1.44	24.35 ± 1.84	0.15 ± 0.14	0.43 ± 0.50
	CYB-short	*0.001*	*0.005*	27.95 ± 1.41	26.80 ± 1.73	0.12 ± 0.11	0.29 ± 0.33
	CYB-long	*<0.001*	*<0.001*	27.49 ± 1.40	26.23 ± 1.66	0.11 ± 0.10	0.28 ± 0.35
	HBB-short	0.064	0.120	34.36 ± 2.13	33.46 ± 2.27	0.17 ± 0.13	0.36 ± 0.45
	HBB-long	0.098	0.107	33.44 ± 2.28	35.58 ± 1.96	0.39 ± 0.69	1.22 ± 3.24

Ct: cycle threshold; *CYB:* Apocytochrome B; *HBB:* Hemoglobin subunit beta; *HV1:* Hypervariable region 1; SD: Standard deviation. *HBB* as nuclear marker vs *HV1* and *CYB* as mitochondrial markers. (*) Just FDR p values comparing genes in cases vs controls by qPCR analysis. Adjusted p values (cn) represent values of comparisons cases vs controls in copy number by dPCR analysis. The italics indicates significant p values.
